# Intestinal Obstruction Due to an Incarcerated Obturator Hernia: A Case Report and Review of the Literature

**DOI:** 10.7759/cureus.51382

**Published:** 2023-12-31

**Authors:** Yogapriya Velumani, Navin Kumar, Karamveer Singh, M Murali Naik, Somprakas Basu

**Affiliations:** 1 General Surgery, All India Institute of Medical Sciences, Rishikesh, Rishikesh, IND; 2 Radiology, All India Institute of Medical Sciences, Rishikesh, Rishikesh, IND

**Keywords:** hernia, hernia in elderly, incarcerated hernia, intestinal obstruction, obturator hernia

## Abstract

An obturator hernia (OH) is a rare form of pelvic hernia in which the abdominal contents protrude through the obturator canal. Malnourished, frail, and multiparous elderly females are at risk of an OH. Preoperative diagnosis of obturator hernia is challenging because of non-specific symptoms. Most of the cases of OH reported in the literature are diagnosed during a laparotomy for acute intestinal obstruction. However, a contrast-enhanced computed tomography (CECT) scan of the abdomen is the best diagnostic investigation for OH. The morbidity and mortality are high because of the associated complications. We report a case of an obturator hernia in a 79-year-old cachectic female with features of acute intestinal obstruction and the usefulness of a CT scan in the preoperative diagnosis. Early diagnosis and treatment are the keys to preventing disastrous complications.

## Introduction

An obturator hernia (OH) is one of the rarest types of pelvic hernias and accounts for 0.04% of all abdominal wall hernias [1]. Ronsil had first described it in 1724 [2]. Malnourished, frail, and multiparous elderly females in their seventh or eighth decade of life are at risk for an OH. The obturator foramen is covered by the obturator membrane and, on either side, by obturator muscles. The obturator nerve and obturator vessels pierce the obturator membrane. This neurovascular bundle is covered by adipose tissue. Loss of these adipose tissues due to malnutrition puts a person at risk for OH. It is associated with high mortality and morbidity (15%-25%), mainly due to delayed diagnosis and associated bowel infarction [3, 4]. An OH is present with small intestinal obstruction in 90% of the cases [3, 4]. Because of the small hernial orifice, strangulation is reported in 50% of the cases of an incarcerated OH.

## Case presentation

A 79-year-old woman presented to the surgical emergency room with complaints of pain in the left thigh followed by lower abdominal pain for 10 days. The pain was of insidious onset, followed by bilious vomiting, abdominal distention, and non-passage of the flatus and feces for three days. She had a history of treatment for pulmonary tuberculosis six months ago. There were no other known comorbidities or surgical history. She had a history of significant weight loss in the last six months. A general physical examination revealed a cachectic-looking woman with a BMI of 17.8 kg/m^2^, a heart rate of 110 beats per minute, and a blood pressure of 90/60 mmHg. Abdominal examination revealed generalized distention with tenderness in the left iliac fossa, and bowel sounds were exaggerated. A digital rectal examination was unremarkable. Routine blood investigations showed elevated white blood cells (WBCs) (40 10*3/uL) and hyponatremia (126 mmo/L); the rest of the blood reports were within normal limits (Table 1).

**Table 1:** Blood biochemistry reports of the patient WBC: white blood cells; Na+: sodium; K+: potassium; AG: albumin and globulin

Test name	Observation result	Reference range
WBC	40 10*^3^/uL	4-11 10*^3^/uL
Hemoglobin	11 g/dL	13-17 g/dL
Serum Na^+^	126 mmo/L	135-145 mmo/L
Serum K+	3.8 mmo/L	3.5-5.1 mmo/L
Blood urea	27 mg/dL	17-43 mg/dL
Serum creatinine	0.50 mg/dL	0.72-1.18 mg/dL
Total bilirubin	0.59 mg/dL	0.1-1.2 mg/dL
Serum total protein	5.5 g/dL	6.6-8.3 g/dL
Serum Albumin	3.3 g/dL	3.5-5.2 g/dL
Serum globulin	2.2 g/dL	2.5-3.2 g/dL
AG ratio	1.5	1.1-2.1

**Figure 1:**
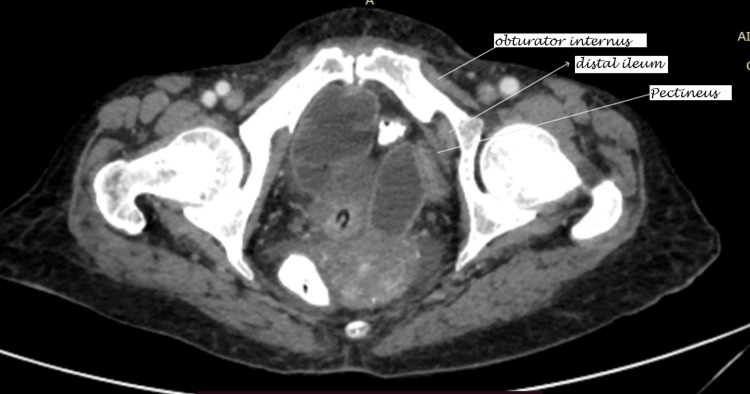
A CECT of the abdomen (axial view) shows an obturator hernia (distal ileum) with dilated small bowel loops. CECT: contrast-enhanced computed tomography

**Figure 2:**
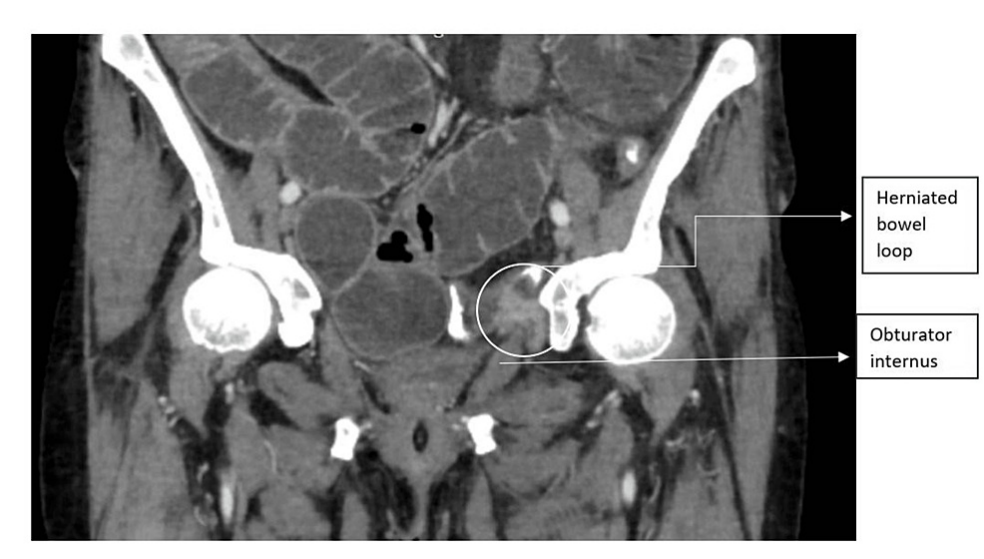
A CECT of the abdomen (coronal view) shows an obturator hernia with dilated small bowel loops. CECT: contrast-enhanced computed tomography

An X-ray of the abdomen revealed dilated small bowel loops. A provisional diagnosis of acute intestinal obstruction was made. She was resuscitated, and contrast-enhanced computed tomography (CECT) of the abdomen and pelvis was done, which showed dilated jejunal and ileal loops with an ileal loop entering through the left obturator foramen (Figures 1-2) and a collapsed large bowel.

**Figure 3:**
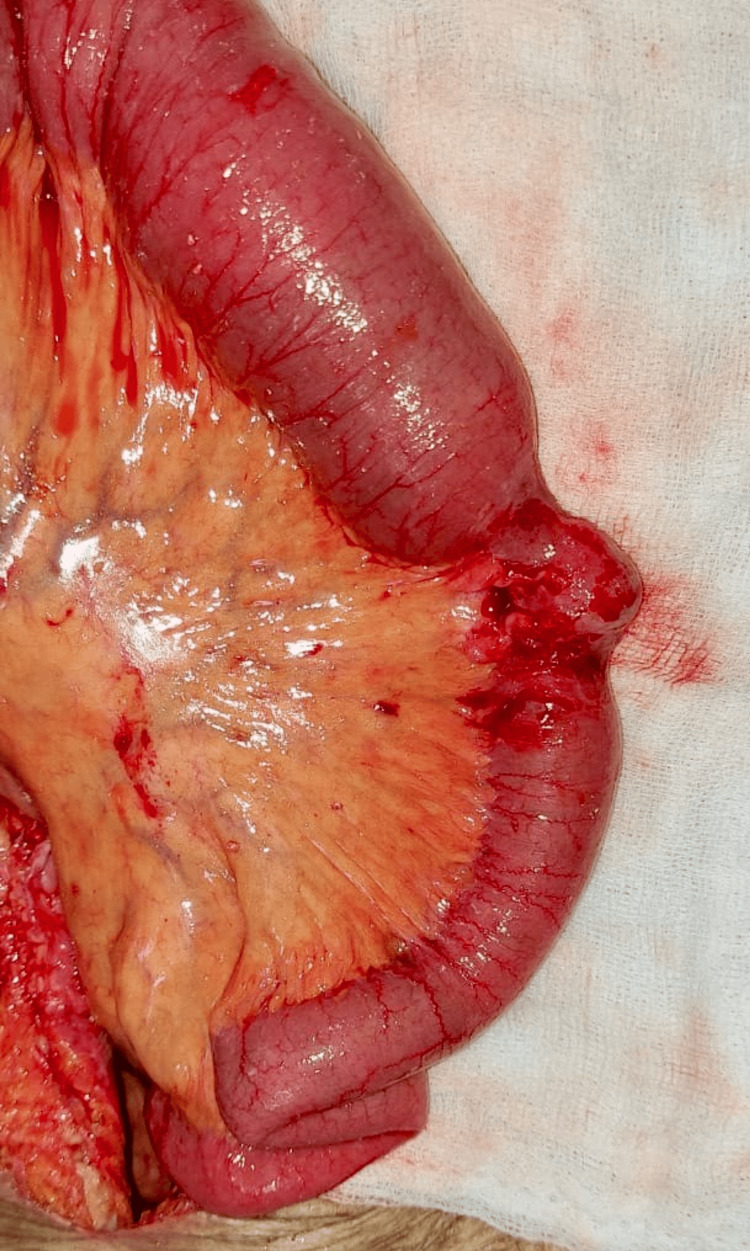
An intraoperative image showing Richter's type of obturator hernia

The patient underwent an emergency exploratory laparotomy. Richter's type of left obturator hernia of the distal ileum intraoperatively without any features of ischemia was noted (Figure 3).

A herniated bowel loop was released, and the OH defect was repaired with an interrupted suture with Prolene 2-0. In the postoperative period, total parental nutrition (TPN) was started on postoperative day (POD) one along with clear liquids as the patient was nutritionally deprived. The patient was allowed an oral diet from POD two. The TPN was gradually weaned off on POD five. The patient had an uneventful postoperative course and was discharged on POD seven. 

## Discussion

An obturator hernia is a rare entity associated with high mortality and morbidity (15%-25%), mainly due to delayed diagnosis and associated bowel gangrene [4]. An OH is more common in females than males because of the wider pelvis and more triangular obturator canal in females [5]. The preoperative clinical diagnosis of an OH is complex, and most patients are diagnosed during surgery for acute intestinal obstruction [6]. Obturator hernia may present as obturator neuralgia or a palpable mass in the upper thigh between the pectineus and adductor longus muscles [7]. Obturator neuralgia may be detected by the Howship-Romberg sign and the Hannington-Kiff sign. Howship-Romberg sign is a characteristic of incarcerated OH in which the patient experiences aggravated ipsilateral groin pain by extension, adduction, and medial rotation of the thigh [8]. It is present in 25%-50% of cases [8]. It is due to compression of the cutaneous branch of the obturator nerve by the content of the hernia. The Hannington-Kiff sign is demonstrated by a loss of the adductor reflex in the presence of a positive patellar reflex.

A CECT scan of the abdomen or an MRI can be done for diagnosis [9]. A CECT scan is diagnostic in identifying the cause of intestinal obstruction with an accuracy of 87%-100% [10]. In the case of an OH, a CECT scan shows the protrusion of the bowel through the foramen between the pubic muscle and obturator externus. It can also detect complications like ischemia and perforation. Richter's type of OH is usually the most common variety because of the small hernial orifice, with the ileum as the most shared content of the hernial sac (50%) [6]. The hernial sac may contain the ileum (50%), omentum, ovary, fallopian tube, or uterus [10]. It frequently occurs on the right side with a right/left ratio of 1.3-5:1 [6]. This is because the left obturator foramen is covered by the sigmoid colon. However, a study by Li et al. (2021) did not find any difference in the sites [11, 12]. Treatment of OH is surgery (open trans-peritoneal or laparoscopic), even in the non-strangulated variety, because of a higher chance of strangulation. A laparoscopic approach should be taken if diagnosed preoperatively, which has the advantage of looking at the contralateral side as well. However, the laparoscopic approach is challenging due to the limited space and dilated small intestine. The management of an OH depends on the size of the defect [13]. Obturator defect <1 cm should be primarily repaired using a non-absorbable interrupted suture, which has a recurrence rate of < 3%. If the defect size is >1 cm, then it can be repaired by hernioplasty with the use of a mesh or with adjacent aponeurosis or muscle, greater omentum, or round ligaments [13, 14]. However, the use of prosthetic material should be avoided due to the high risk of infection. There are incidences of delayed intestinal necrosis due to incarcerated intestinal obstruction. The intestinal resection rate in OH is 25%-50% because of the development of gangrene [15]. In the presence of intestinal obstruction or perforation and severe comorbidities, an emergency laparotomy should be performed through a midline incision.

## Conclusions

An obturator hernia is an unusual and rare entity that has high rates of mortality due to delays in the diagnosis and development of intestinal gangrene. An OH should be kept as a differential diagnosis in an emaciated elderly female patient with acute intestinal obstruction. A CECT scan is the diagnostic modality of choice for OH. Early intervention with primary repair of OH without a mesh is safe because of the risk of delayed intestinal necrosis due to an incarcerated intestinal obstruction.
